# A First Generation BAC-Based Physical Map of the Asian Seabass (*Lates calcarifer*)

**DOI:** 10.1371/journal.pone.0011974

**Published:** 2010-08-05

**Authors:** Jun Hong Xia, Felicia Feng, Grace Lin, Chun Ming Wang, Gen Hua Yue

**Affiliations:** Molecular Population Genetics Group, Temasek Life Sciences Laboratory, National University of Singapore, Singapore, Singapore; Lund University, Sweden

## Abstract

**Background:**

The Asian seabass (*Lates calcarifer*) is an important marine foodfish species in Southeast Asia and Australia. Genetic improvement of this species has been achieved to some extent through selective breeding programs since 1990s. Several genomic tools such as DNA markers, a linkage map, cDNA and BAC libraries have been developed to assist selective breeding. A physical map is still lacking, although it is essential for positional cloning of genes located in quantitative trait loci (QTL) and assembly of whole genome sequences.

**Methodology/Principal Findings:**

A genome-wide physical map of the Asian seabass was constructed by restriction fingerprinting of 38,208 BAC clones with SNaPshot HICF FPC technique. A total of 30,454 were assembled into 2,865 contigs. The physical length of the assembled contigs summed up to 665 Mb. Analyses of some contigs using different methods demonstrated the reliability of the assembly.

**Conclusions/Significance:**

The present physical map is the first physical map for Asian seabass. This physical map will facilitate the fine mapping of QTL for economically important traits and the positional cloning of genes located in QTL. It will also be useful for the whole genome sequencing and assembly. Detailed information about BAC-contigs and BAC clones are available upon request.

## Introduction

A physical map is essential to support fine mapping of quantitative trait loci (QTL) for economically important traits and to facilitate positional cloning of genes located in QTL [1]. A genome-wide physical map is also a natural component of large genome sequencing endeavors [2], [3]. The map can not only provide the start point for the clone-by-clone genome sequencing approach and assembly assessment, but also be used to validate and improve the contig layout of existing whole-genome shotgun sequence assemblies [1], [2], [4]. Currently, physical mapping of genome with large-insert clones such as Bacterial Artificial Chromosomes (BACs) and fosmids by fingerprint analysis is becoming an active area of genomics research [5].

Several gel-based restriction fingerprinting methods have been used for physical mapping of some genomes from BACs, such as *Caenorhabditis elegans* and *Saccharomyces cerevisiae* [6], human [7], *Arabidopsis thaliana* [8] and rice [9]. However, the fingerprints based on the acrylamide and agarose gel-based methods have some disadvantages such as low information, imprecise sizing, lack of efficiency and demands of considerable skill [10]. Compared to the gel-based fingerprinting methods, the application of capillary electrophoresis in fingerprinting significantly increased the efficiency and accuracy of quality of a physical map construction [11]. The fluorescent-based high-information-content fingerprinting (HICF) technique taking the advantage of automated capillary DNA sequencing instruments have recently been developed for physical mapping of maize [10], tilapia [12], catfish [13] and rainbow trout [14]. These studies indicated that the HICF technique could be a high-resolution and high-throughput fingerprinting.

The Asian seabass *Lates calcarifer* (also called barramundi in Australia) is a catadromous fish species that belongs to the family Latidae of order Perciformes [15]. This species is one of the most well known salt and freshwater foodfish species and is widely distributed in the Indo-West Pacific region from the Persian Gulf, through Southeast Asia to Northern Australia [15]. This fish has been cultured in Thailand, Malaysia, Singapore, Indonesia and Australia. It is of large commercial importance with a global annual production 400,000 metric tons according to Food and Agriculture Organization statistics [16], [17]. To facilitate the breeding of this species, some genomic tools such as microsatellites [18], [19], single-nucleotide polymorphisms (SNPs) in genes [20], a linkage map containing 240 microsatellite markers and genes [18], a BAC and several cDNA libraries [17], [20] have been developed. Microsatellites have been used to study genetic diversity in the wild and in hatcheries [19], [21] and the linkage map has been applied to mapping QTL for growth traits [19], [22]. However a physical map is still lacking, although it is essential for fine mapping of QTL controlling economically important traits, positional cloning of genes located in QTL and sequencing and assembling the whole genomes. Here, we describe a first generation BAC-based physical map of the Asian seabass constructed using SNaPshot HICF Fingerprint Contigs Map (FPC) techniques.

## Methods

### Ethics Statement

All handling of fish was conducted in accordance with the standard operating procedure set up by Institutional Animal Care and Use Committee (IACUC) of the Temasek Life Sciences Laboratory, Singapore. The IACUC approval number is TLL (F)-002-09.

### BAC library fingerprinting

One BAC library was constructed for the Asian seabass [17]. BAC clones from each 384-well plate were inoculated in four 96-well plates with 1.4 ml of 2x YT medium containing 12.5 µg/ml chloramphenicol. The clones were incubated at 37°C for 22–24 h with shaking at 250 rpm. BAC DNA was isolated using a modified alkaline lysis method [23]. The DNA was then resuspended with 30 µl ddH_2_O overnight at 4°C. Restriction fingerprints were obtained following the approach of Luo et al. [24]. Briefly, the isolated DNA was digested with H*ae*III, E*coR*I, X*ba*I, X*ho*I, B*amH*I (New England Biolab, Ipswich, MA) for 4 hours at 37°C. Fragments were labeled with the SNaPshot kit (Applied Biosystems, Foster City, CA) at 65°C for one hour according to the manufacture's recommendation. The resulting labeled fragments were precipitated with sodium acetate (pH 5.2) and prechilled ethanol (−20°C) and then kept at −80°C for 10–15 min or overnight at −20°C. Dried DNA was dissolved in 10 µl of Hi-Di formamide (ABI NO. 4311320) and 0.1 µl of GeneScan-500 LIZ size standard (ABI No. 4322682) for at least 1 hour at 4°C. The samples were resolved on a 3730xl DNA Analyzer (Applied Biosystems, Foster City, CA). Reproducibility of fingerprinting techniques was assessed by repeatedly fingerprinting randomly selected clones (with more than 30 bands) and using Genoprofiler software [25].

### BAC contig assembly

The restriction fragments were sized against the internal size standard (GeneScan-500 LIZ) using ABI GeneMapper 3.5 software package (Applied Biosystems, Foster City, CA). The exported data files were then analyzed with GenoProfiler software to remove the peaks from background noise, vector bands and potential contaminations from neighboring wells. The files generated were used in the contig assembly using FPC software version V8.9 [26], [27]. Considering the lower resolution of the bands less than 75 bp [24], contigs were assembled only from bands ranging from 75 to 500 bp. Based on the approach described in Meksem and Kahl [28] every size was multiplied by a factor of 10, as a result, the gel length was set at 17000 bp. As we observed a maximum standard deviation of 0.19 bp for vector bands (n = 300) in the experiment the tolerance was set to 4. Due to an average insert size of 98 kb [17] and average valid bands of 72 per clone determined in the experiment for this BAC library the estimated band size was set at 1361 bp. The very low initial cutoff values of 1e^−45^ was used in order to limit the number of questionable clones (Q-clones) in contigs. The ‘Best of’ function was set to 60 builds. Contigs with more than 10% Q-clones were reduced by running the DQer with a stricter cutoff by setting the value of DQer function to 10% and the step value to 5. Then, the stringency was decreased from 1e^−45^ to 1e^−15^ with the ‘Ends to Ends’ auto merge function. Finally, the ‘keyset to FPC’ function was used and the stringency was adjusted to 1e^−25^ with a minimum of 2 ends matching.

### Examination of the reliability of the assembly of BAC clones

#### SNP markers and BAC library screening

Expressed sequence tags (ESTs) showing SNPs in contigs of the Asian seabass cDNA libraries were used to develop primers for BAC library screening. Briefly, the EST sequences were aligned with fugu and zebrafish genomic sequence database in GenBank. Primer sites in conserved exon regions were identified and primer pairs allowing PCR amplification of an intron-spanning fragment were developed. PCR reactions for primer tests were performed in 25 µl PCR volume containing 10 ng BAC DNA, 1×PCR buffer, 100 µmol of each dNTPs, 0.2 µmol forward primer, 0.2 µmol reverse primer and 1 U of Taq DNA polymerase (Finnzymes, Espoo, Finland) on a thermal cycler (Bio-Rad, Hercules, CA,USA) with the following cycling profile: one denaturation step for 2 min at 95°C was followed by 35 cycles of 30 sec at 94°C, 30 sec at annealing temperature and 45 sec at 72°C. The final step was a prolonged extension of 5 min at 72°C. PCR products were resolved on 2% agarose gel and visualized by ethidium bromide staining. Finally 113 primer pairs were obtained (Table S2).

For BAC library screening by PCR, pools of the bacterial cultures were constructed as Wang et al. (2008) [17]. Briefly, the library was divided into 11 superpools each consisting of 12 plates of 384-wells. Each superpool was divided into 48 pools each consisting of one 96-well plate of BAC clones. With this method positive clones could be identified by PCR in a sequence of three experiments.

#### Development of BAC end sequence markers and overlapping analysis of adjacent clones by PCR

For testing the assembly reliability of the physical map 14 contigs with a size ranging from 269 to 1099 CB units were randomly selected for contig reliability test. A total of 96 clones representing the contigs were identified using the minimum tiling path (MTP) method for BAC end sequencing and primer development. BAC DNA was isolated using a modified alkaline lysis method [23]. The isolated DNA was sequenced in both directions with primers pcc1BAC_R (CTCGTATGTTGTGTGGAATTGTGAGC) and pcc1BAC_F (GGATGTGCTGCAAGGCGATTAAGTTGG) using BigDye chemicals and an ABI 3730xl sequencer (Applied Biosystems, Foster city, CA). Firstly primer in one end region of MTP clones was developed and used for identification of overlapping relationship of adjacent clones. For adjacent MTP clones with unidentified overlap, new primers were developed based on the opposite end sequences. Primers used for identification of contig reliability by a PCR approach are presented in Table S3. PCR for each primer pair was performed as above.

## Results and Discussion

### Fingerprinting and contig assembly

A BAC library was constructed for the Asian seabass and consisted of 49,152 clones with an average insert size of 98 kb, representing 6.9-fold haploid genome coverage [17]. To develop the whole-genome physical map, a total of 38,208 clones from the BAC library were fingerprinted with SNaPshot HICF FPC technique [24]. Out of 38,208 clones, 35,265 (92%) were analyzed with FPC software after removing the clones not meeting our quality standards. An average of 72 valid bands per clone was detected and each band, on average, represented a ∼1.4 kb fragment of a BAC clone. The summary of the physical map data is presented in Table 1.

**Table 1:** Summary of the first generation BAC-based physical map of the Asian seabass (*Lates calcarife*r).

Number of clones fingerprinted	38,208
Number of clones used for FPC analysis	35,265
Mean insert size (kb)	98
Average valid bands per clone	72
Number of singletons	4,811
Number of contigs	2,865
2 clones	207
3–9 clones	1,476
10–24 clones	981
25–49 clones	183
50–99 clones	17
100–199 clones	1
Q-contigs	696
Qs< = 10%	690
Qs>10%	6
Q-clones	1,387
Number of clones in contig	30,454
Physical map length of the contigs (kb)	665,227
Longest contig (kb; CTG588)	1,478
Genome coverage of fingerprinted clones	5.3 fold
Genome coverage of clones used in map	4.9 fold
SNP markers in the physical map(totally used markers)	107(113)

A total of 30,454 BAC clones were assembled into 2,865 contigs and 4,811 remained singletons. Based on the average size (98 Kb) of BAC clones in our BAC library [17], the 35,265 clones summed up to 3,455.97 Mb. Since the genome of *L. calcarifer* is ∼700 Mb [29], [30], these clones cover 4.9- fold of its haploid genome. This genome coverage is sufficient for construction of a first generation physical map.

**Figure 1:**
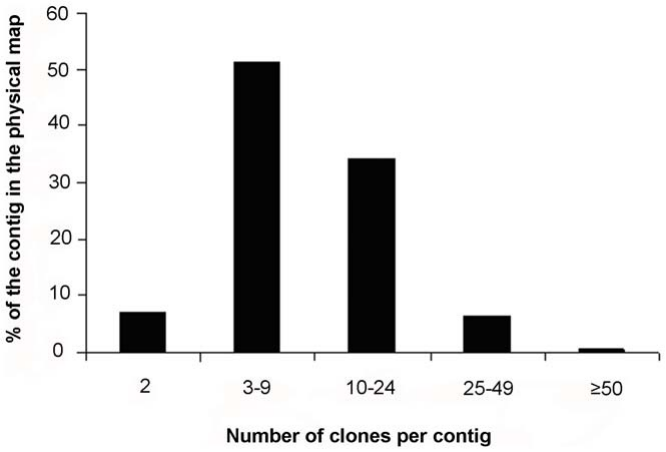
Distribution of contigs of various sizes in the BAC-based physical map of the Barramundi. The ‘%’ value showed the ratio of contigs of various sizes to the total contigs (2865) in the physical map.

**Figure 2:**
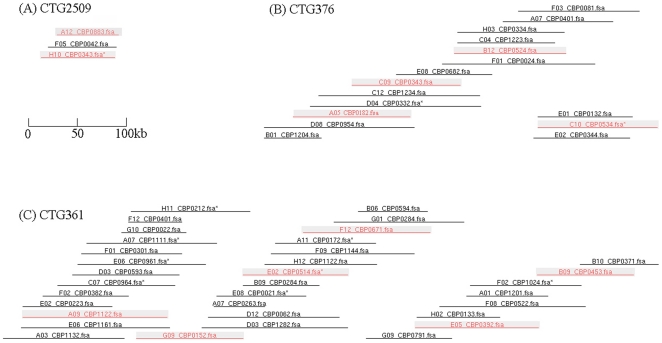
Example of three contigs in the BAC-based physical map of the Barramundi genome. One contig (CTG2509) with 4 clones (one buried clone indicated by a star at the BAC-end), one (CTG376) with 20 clones (4 buried clones) and one (CTG361) with 42 clones (8 buried clones) are presented. These contigs represent a general organization of the contigs with different sizes (clones per contig) in the BAC-based physical map. The identified MTP BAC clones in each contig for BAC end sequencing are shown in gray background color. The contigs are drawn to scale.

The longest contig of the map (CTG588) spanned 1,478 kb in physical length and contained 75 clones. A total of 201 contigs contained 25 or more BAC clones; 981 included 10–24 clones and 1476 contained 3–9 clones. The contig size (clones per contig) distribution is shown in Figure 1 and Figure 2. More than half (51.5%) of the contigs was consisted of 3–9 clones. The estimated average contig size for the physical map was ∼232 kb (range: 44 to 1,478 kb). The physical length of the assembled contigs summed up to ∼665 Mb, being slightly shorter than the estimated genome size (700 Mb) of the Asian seabass [29], [30]. This result suggested that some regions of the Asian seabass genome were poorly represented in the library and there were still gaps that needed to be filled in future. Previous study showed that gaps mainly existed in the heterochromatic regions and it was time-consuming to close the gaps by the conventional chromosome walking approach because of repetitive sequences [31]. Thus, in order to develop a genome-wide physical map of a high-genome coverage for the Asian seabass, it is necessary to develop several individual source BAC libraries with different enzymes as suggested by Chang et al. [31] and Tao et al. [32].

A drawback to HICF methodology was the large number of questionable clones (Q-clones) that were generated, possible caused by inconsistency in enzyme digestion [10], [33], contamination and doubled peaks (approximately 5% of HICF peaks) and possible chimeric contigs [10]. In this study, a reasonably stringent cutoff (from 1e^−45^ to 1e^−15^) and the function DQer were used to break up all contigs containing 10% Q-clones after each merge. The false-positive merges in our final map were reduced greatly. FPC identified a total of 1,387 Q-clones in our assembly, accounting for the 3.9% of the fingerprinted clones used for FPC analysis, which was much less than those of reported maps such as Nile tilapia (8.9%) [12] and channel catfish (7.3%) [13] but higher than that in rainbow trout assembly (1.4%) [14]. However the fraction of contigs with Q-clones in this assembly (24.3%) was higher than tilapia (7.5%) [12], rainbow trout (19.4%) [14] and channel catfish (15.5%; Xu et al.) [34]. This suggested that a few clones could have been placed in the wrong contigs. The distribution of Q-clones in contigs of the BAC-based physical map of the Asian seabass is shown in Table 2. The majority of contigs (75.7%) were free of Q-clones. Only 5.3% contigs had ≥3 Q-clones. The possibility of Q-clone in each contig of the assembly was very low, as reflected by the ratio of Q-clones/clones as well (Table 2).

**Table 2:** Distribution of Q-clones in contigs of the BAC-based physical map of the Asian seabass (*Lates calcarifer*).

*Q-clones/contig*	*Number of contigs*	*Percentage (%) of all contigs*	*Clone in contig*	*Q-clones/clone*
0	2169	75.7	17233	0
1	432	15.1	6205	0.07
2	124	4.3	2522	0.1
3	52	1.8	1398	0.08
4	36	1.3	1010	0.14
5	19	0.7	549	0.17
6	8	0.3	288	0.17
7	7	0.2	243	0.2
8	8	0.7	371	0.17
9	4	0.1	225	0.14
≥10	6	0.2	410	0.28

### Contig reliability

Before fingerprinting of a large number of BAC clones from the library we measured reproducibility of fragment sizes of the same clone by repeatedly fingerprinting BAC clones. Our results showed that the reproducibility of the overall band pattern was around 0.84 (n = 300), similar to that (0.85) of the channel catfish [13] and higher than that (0.75) of the maize [10]. We also observed a maximum standard deviation of 0.19 bp for vector bands (n = 300) in experiments, being smaller than that of the Nile tilapia physical map (0.5 bp) [12] and the channel catfish map (0.4 bp) [13]. Hence, the sizing of fragments in this study is highly reliable.

If one single copy gene or fragment of the genome is sequenced, one pair of primers designed from this sequence can be used for screening all clones individually in the BAC library developed from the genome by PCR. The clones with the PCR product of correct size should contain the same single copy gene or fragment of the genome. Therefore, if the map contigs were reliable, the positive BACs screened with a single-copy marker should originate from the same region of the genome and all be assembled to a segment of a single contig based on the overlap among clones [1], [32]. We screened around one third of the clones from the BAC library [17] using 113 newly developed SNP markers derived from our EST library. A total of 236 positive clones were detected for the 113 SNP markers with a number of targeted clones ranging from 1 to 11 clones for each marker (Table S1 and S2). Our data showed that the positive BACs screened by each of 61 (54%) of the markers located in a single contig. The localization of the positive clones selected with each marker at two or more contigs could be multiple copies of the DNA markers in the genome or contig assembly errors [32]. Since little information was available for these loci in the Asian seabass genome it was difficult to investigate whether the loci were duplicated or not. In the human genome it was estimated that around 38% protein genes were duplicated [35] and in Arabidopsis ∼23% of the proteomes were duplicates [36]. When similar duplications in the human and Arabidopsis protein genes were applied to the Asian seabass, the single-copy marker number used in this study was estimated to be around 70–87. Thus, an estimate of around 70–87% of the positive BACs screened for each marker should be located to a single contig in this study. The ratio was similar to those in other studies, such as Tao et al. (∼74%) [32] and Ren et al. (∼82%) [1]. Since an extra genome duplication was occurred in the fish lineage after it diverged from the tetrapod lineage [37]–[40], the duplication of fish genes should be higher than those of human and Arabidopsis genes. In addition, the positive clones from more than one contig detected by a high percentage (46%) of the presumed single copy markers may also indicate that some of the positive contigs actually overlap, but that this was not identified using the stringent criteria of contig assembly in this study. However, analysis at less stringent criteria would have a much higher risk of wrong contigs. Therefore our map contigs should be reliable. We are planning to anchor these SNP markers to the medium density linkage group of Asian seabass [17] in the future.

Overlapping BAC clones in a given contig can also be identified using a BAC end sequence-based PCR approach. Two BACs yielding the same size of PCR product using a particular pair of PCR primers developed from the end sequences of the clones will be assumed to carry an overlap. For testing the assembly reliability of the physical map, 96 clones representing 14 randomly selected contigs with a size ranging from 269 to 1099 consensus band (CB) units were identified using the minimum tiling path (MTP) method for BAC end sequencing (BES; Table 3). Finally, a total of 106 primer pairs in end regions of MTP clones were developed and used for identification of pairwise overlapping relationship of adjacent clones (Table S3). Out of the 82 overlaps for the 14 contigs, 79 (96.3%) were identified. The remaining 3 unidentified overlaps (3.7%) existed in CTG322 (D04_CBP0882 - C08_CBP1271), CTG588 (F07_CBP0533 - F12_CBP1184) and CTG686 (G03_CBP0093 - C07_CBP0191) (Table 3). The contig assembling error was estimated at 4% [10] and 5% [13]. Thus, the false joins in our contigs were similar to the above reported.

**Table 3:** Summary for the MTP BAC clones in selected 14 contigs used for BAC end sequencing and assessment of contig reliability by a PCR approach.

*Contig name*	*MTP clones identified in the contig*	*CB units*	*Contig assembly completely validated*
CTG25	D04_CBP0583 B07_CBP1064 F05_CBP0443	246	yes
CTG53	G05_CBP0943 B03_CBP0262 E01_CBP0512 B05_CBP5000 B11_CBP0233 D08_CBP1203 D07_CBP0944 B05_CBP0372 G07_CBP0384 H10_CBP0063	632	yes
CTG125	B12_CBP0021 B07_CBP1134 H03_CBP0792 C05_CBP0432 B08_CBP0673 F07_CBP0244 G09_CBP1111 B11_CBP0323 C12_CBP0153	701	yes
CTG215	D03_CBP0372 E09_CBP0954 F08_CBP1214 H07_CBP0933	355	yes
CTG231	B03_CBP0481 H02_CBP1041 B03_CBP0252	301	yes
CTG322	C11_CBP1021 D09_CBP0604 D04_CBP0882 C08_CBP1271 C04_CBP1044 G11_CBP1172	598	no
CTG372	C04_CBP1022 A03_CBP0152 G01_CBP0312 B11_CBP0623 H12_CBP0642 E04_CBP1133	559	yes
CTG491	D01_CBP1201 H08_CBP1272 E05_CBP0074 E08_CBP0134 C04_CBP0482 G04_CBP1013 H11_CBP0351 G07_CBP1071	602	yes
CTG588	F11_CBP0683 A12_CBP1041 B07_CBP0313 C04_CBP1274 C06_CBP0434 D06_CBP5000 H05_CBP1042 E06_CBP0231 F07_CBP0533 F12_CBP1184 C10_CBP0261 C02_CBP1234 H06_CBP1241	1099	no
CTG598	B08_CBP0692 E01_CBP0502 C02_CBP0174 C01_CBP0314 E02_CBP0502 E08_CBP0052 B05_CBP1181 B02_CBP0582 G03_CBP0012 E02_CBP1174	665	yes
CTG686	C03_CBP0553 D04_CBP0101 G03_CBP0093 C07_CBP0191 B09_CBP0381 F12_CBP0533 D11_CBP0654 D10_CBP1281 A02_CBP0333	650	no
CTG869	C02_CBP0643 D06_CBP0624 D12_CBP1094 G06_CBP0332 G02_CBP0534 B02_CBP1154 B03_CBP0263	475	yes
CTG921	G04_CBP0074 A10_CBP1191 E11_CBP0573 G06_CBP1134 A05_CBP1194	493	yes
CTG1210	H08_CBP1171 C10_CBP0023 G01_CBP0283	269	yes

A total of 3 putative overlaps in CTG322 (D04_CBP0882 - C08_CBP1271), CTG588 (F07_CBP0533 - F12_CBP1184) and CTG686 (G03_CBP0093 - C07_CBP0191) were unidentified by a PCR approach.

Our analysis showed that the contigs constructed in this study were reliable. However, our studies also suggested that some chimeric contigs still existed in our assembly resulting from repetitive bands or contamination; resultantly some of the contigs could have been mis-joined. Better computational tools for identifying specific Q-clones in each contig and additional fingerprinted data of individual source BAC libraries with different enzymes would be used to improve the quality of the physical map in the near future.

### Conclusion

We have constructed a first generation physical map for the Asian seabass and the reliability of the resulting map was confirmed by several validation methods. This map could provide a platform for fine mapping of QTL for economically important traits and positional cloning of genes located in QTL, and accelerating genome studies in physical map-assisted genome sequence assemblies. We also expect the BAC-based physical map of the Asian seabass will be used in the comparative analysis of genomes in the near future. Detailed information about BAC-contigs and BAC clones are available upon request.

## Supporting Information

Table S1 Positive clones in Asian seabass BAC library screened using SNP markers and their locations in Asian seabass physical map.
(0.05 MB XLS)

Table S2 SNP marker primers used for identification of contig reliability.
(0.04 MB XLS)

Table S3 The primer sequences used for identification of contig reliability by a PCR approach.
(0.06 MB PPT)
